# Usability evaluation of the *Agente Escuta* application: translational research

**DOI:** 10.1590/2317-1782/20232022149en

**Published:** 2023-09-15

**Authors:** Alice Andrade Lopes Amorim, Kátia de Freitas Alvarenga, Lilian Cássia Bórnia Jacob, Eliene Silva Araújo

**Affiliations:** 1 Programa Associado de Pós-graduação em Fonoaudiologia, Universidade Federal do Rio Grande do Norte - UFRN - Natal (RN), Brasil.; 2 Faculdade de Odontologia de Bauru - FOB, Universidade de São Paulo - USP - Bauru (SP), Brasil.

**Keywords:** User-Centered Design, Health Education, Telemedicine, Community Health Workers, National Policy for Hearing Health Care

## Abstract

**Purpose:**

To evaluate the usability and satisfaction of users with the interface of the ‘*Agente Escuta*’ application, in addition to identifying problems and possibilities for improvement.

**Methods:**

Descriptive exploratory translational study, characterized by a usability test with a quantitative and qualitative approach, subdivided into three stages: (I) prior evaluation of usability by 10 judges, including students, primary care professionals, professors and researchers in Information Technology and Speech Therapy; (II) evaluation of the application by the target audience, that is, community health agents from six municipalities in Rio Grande do Norte; (III) evaluation of the satisfaction of the agents who used the application in their work routine. The System Usability Scale and the Net Promoter Score were used, in addition to a qualitative evaluation of the opinions.

**Results:**

Usability was rated as excellent by judges, regardless of category. In the evaluation by community health agents, usability was considered good and there was no effect of the city of origin. It was found that the perception of the judges and the target audience were different, with a lower score for the participants in the second stage. However, most would give positive publicity to the product. The heuristic with the highest score was ease of memorization and participants in the third stage were interested in continuing to use the tool in practice, even after the end of the study.

**Conclusion:**

The *Agente Escuta* prototype showed good usability and satisfaction and aspects that could be improved in future solutions were identified.

## INTRODUCTION

The expansion and improvement of Primary Health Care (PHC) are viewed as the primary initiatives to foster qualitative changes in healthcare for the coming decades worldwide^(1)^. In Brazil, the National Primary Care Policy (NPCP) provides for the articulation of institutions in partnership with the health departments to offer permanent and continuing education for PHC professionals^(2)^.

When specifically considering hearing health, the World Health Organization (WHO) report in 2021 highlights alarming projections regarding the prevalence of hearing loss, with an estimate that 900 million people may have some degree of hearing loss by 2050. The document reiterates the number of preventable causes, as well as the annual global cost of US$ 750 billion for untreated hearing loss^(3)^. Thus, the importance of PHC in hearing health is undeniable, as well as the need to develop tools that reach different locations in the country.

Considering this context, there has been considerable growth in innovative programs and technologies aimed at strengthening this level of care. Technological solutions in telehealth began to be developed for health professionals, such as Community Health Workers (CHW), to overcome barriers to accessing hearing health care, using smartphones, tablets, computers and other portable devices^(4)^. Thus, innovations in health education based on mHealth (mobile health) are already a reality and have grown exponentially in the last five years in other countries^(5,6)^.

In Brazil, the trend has not been different. Currently, there is the “*Informatiza APS*” program, which is part of the Ministry of Health's digital health strategy^(7)^, and the e-SUS AD^(8)^ and e-SUS territory^(9)^ apps aimed at facilitating the work process of local CHWs.

In South Africa, since the 2000s, mHealth technologies have been used in the hearing health program^(10)^. These validated mHealth technologies enabled PHC to perform hearing screenings using automated tests with a smartphone interface. Considering this, the CHWs began to conduct hearing screening for children in different community contexts, including home visits and monitoring of early childhood development^(11)^.

Evaluations of this model have proved that the CHW can be trained to screen children reliably and time-efficiently. However, there are challenges in this program, such as the levels of environmental noise during the exams, which must be adequate so that they do not affect the reference rates of the hearing screening. Another challenge lies in monitoring the quality of the hearing screening conducted by CHWs^(11)^.

Therefore, the usability evaluation of mobile applications is a strategy to ensure that interactive systems are adapted to users and their tasks, and that there are no negative quotients regarding their use. The objective of a usability evaluation is to verify the degree to which a system or product is effective, that is, how well the system fulfills the tasks for which it was designed; efficient, that is, how many resources, such as time or effort, are required to use the system to perform the tasks for which the system was designed; and, finally, whether it favors positive attitudes and responses from would-be users^(12)^.

However, the ‘*Agente Escuta*’ application, developed in this study, was designed to assist PHC professionals in monitoring the development of hearing and language, based on developmental milestones. Another objective of the app is to promote continuing education in hearing health for CHWs in an interactive way.

However, as it is an unprecedented app, there are no previous studies on its usability or characterization of its implementation in the Brazilian context. Therefore, the objective of the present study was to verify the usability and satisfaction of users with the interface of the ‘*Agente Escuta*’ application, in addition to identifying problems and possibilities for improvements.

## METHODS

This is an exploratory translational study with a prospective descriptive design. It was carried out through a usability test of the ‘*Agente Escuta*’ app with a quantitative and qualitative approach to identify difficulties and possible improvements in the tool.

The study was structured in three stages, namely: (I) prior evaluation of usability by 10 judges, including students, primary care professionals, professors and researchers in Information Technology and Speech Therapy; (II) evaluation of the application by the target audience, CHWs from six municipalities in Rio Grande do Norte (RN), Brazil; (III) evaluation of the satisfaction of the CHW who used the application in their work routine.

Following the Guidelines and Regulatory Standards for Research Involving Human Subjects (Resolution 466/12), the study was only initiated after approval by the Institution's Research Ethics Committee (number process 4.695.580) and upon signing of the Free Consent Form, providing all necessary explanations regarding study participation.

### Context

In partnership with the Metrópole Digital Institute of the Federal University of Rio Grande do Norte (MDI/FURN), recognized for its projects with an interface in health, the prototype of the application was developed over a period of one year, between 2020 and 2021. The first version of the tool was developed with an average of 15,000 lines of code and predominantly in Javascript language, with the back-end and front-end of the mobile version and the dashboard of the web version being programmed. The software registration at the National Institute of Industrial Property (NIIP) was issued in November/2021 with protocol number BR512021002590-3.

The app works on two main axes: continuing education in hearing health, and monitoring of the development of hearing and language in children from zero to 12 months of age. In the continuing education axis, tabs are available for content review, namely: the most frequent doubts about hearing health raised by the CHWs while using the app, a flowchart with the development milestones in the first two years of life, and *Escuta* Game, presented on the home screen, which contains daily situations related to hearing health.

**Figure 1:**
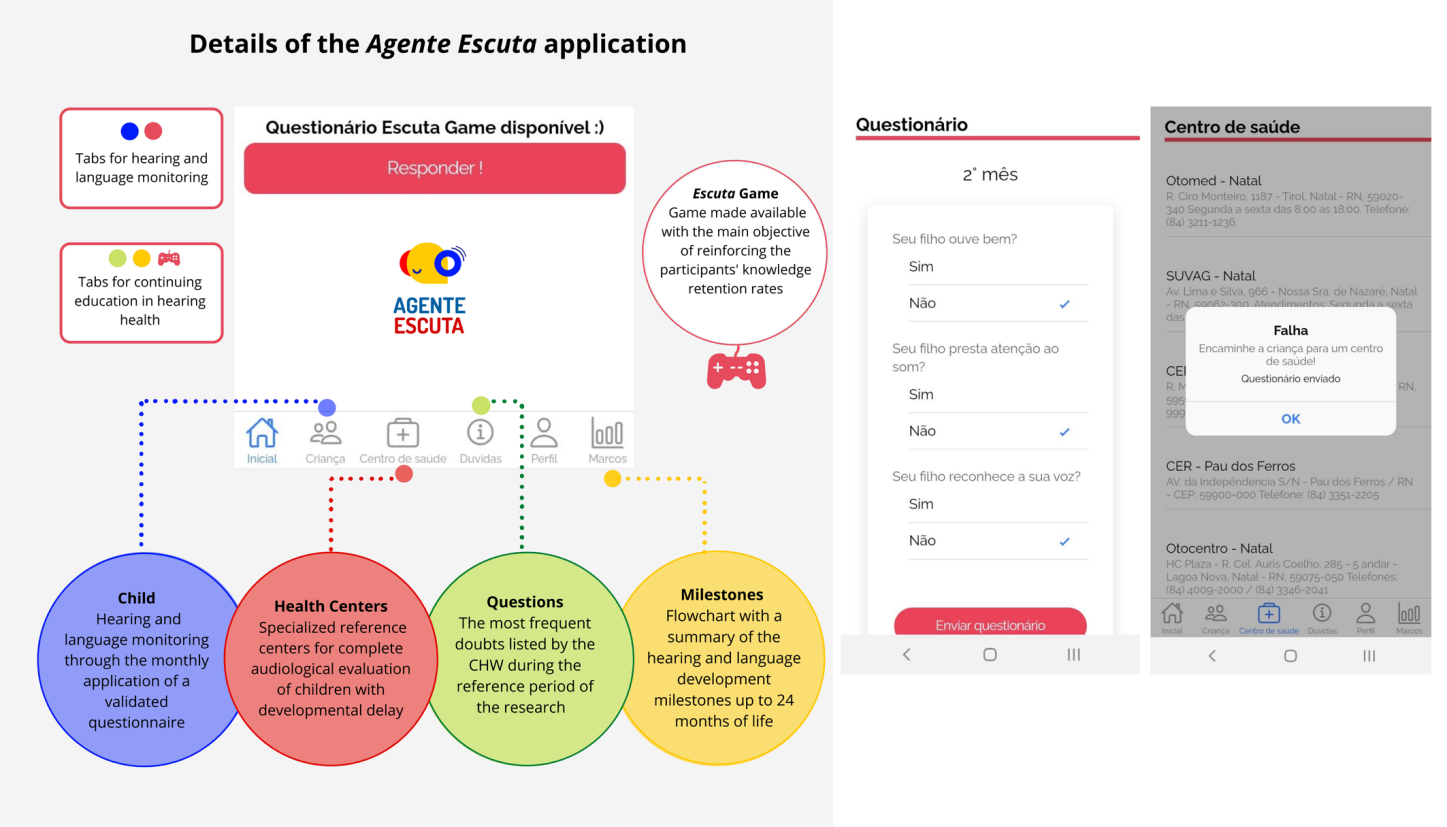
Infographic summarizing the features of the *‘Agente Escuta’* and screen of the follow-up questionnaire with message indicating failure, in the official language of the application (Portuguese)

For the construction of its functionalities, a survey was carried out of already validated tools, such as guidance booklets based on development milestones, questionnaires and international and national guidelines^(13-17)^. Figure 1 summarizes the app features.

**Caption:** CHW = Community Health Workers; Inicial= Home page; Perfil= Profile; Questionário Escuta Game disponível= ‘Listening game’ quiz available; Responder= answer; Questionário= questionnaire; 2º mês= 2^nd^ month; Centro de saúde= Health Centers; Falha= fail

In terms of monitoring hearing and language, the application provides questions regarding the age of the registered child^(17)^. When answered, the specific questionnaire for each child will only be available again in the following month, with the subsequent age group, which reduces the chances of a duplicate response within the month. According to the “failure” criterion proposed in the follow-up questionnaire^(17)^, the app automatically refers the user to the “health centers” tab, where all the hearing health centers in the region are listed so as to facilitate the referral for audiological evaluation.

All this information is sent to the MySQL database, which is a relational database connected to a private server with technology based on cryptography, and can be continuously accessed only by the administrator profile (ADM), who, in this case, are the researchers in charge.

In the ADM version, information is available on the number of questionnaires applied, successes and errors in the game, results of each child monitored, location, risk indicators for hearing loss of the registered children, whether the NHS was performed, and results obtained.

### Participants

For the prior evaluation of the usability of the app, two judges from each category, not involved with the development of the app or in other stage of the research, were invited, namely: undergraduate and graduate students in Speech Therapy, CHW, professors and researchers in Technology of Information and in Speech Therapy. The 10 evaluators were selected based on their professional activities, history of developing mobile applications with a health interface and/or participation in university internships with actions directly linked to the promotion of children's hearing health in PHC.

To obtain a paired analysis, it was decided to include two participants in each category. A third judge would be invited if the total scores of the usability scale between the two evaluators showed more than 20 points of difference in the individual evaluation. A total of three male and seven female judges, aged between 21 and 48, participated in this first stage.

In the second stage of the research, the usability of the app was evaluated by CHWs interested in using the tool in their daily practice. The definition of the minimum sample size was based on the estimate that a single user is capable of finding, on average, 31% of the usability issues, five users are enough to identify 85% of the issues and that, when carrying out the test with 15 users, approximately 100% of the problems can be identified^(18)^. Thus, the sample size of 15 CHWs was defined, with a minimum representativeness of five users in each mesoregion of RN.

To guarantee greater representativeness of the general context of the RN, it was decided to select the municipalities through a draw by conglomerates. For the draw, the four mesoregions of RN were considered, namely: Agreste Potiguar, Central Potiguar, East Potiguar and West Potiguar. Except for Natal, which was selected because it is the state capital, the municipalities of each mesoregion were randomly selected, namely: Caicó, Upanema, Santa Cruz, São Miguel do Gostoso and João Câmara.

In agreement with the municipal health secretaries, for each municipality drawn, Primary Health Care Unit (PHCU), inserted in neighborhoods with a greater number of children aged between zero and 12 months, was selected.

Inclusion criteria for the first two stages included: owning a smartphone with the Android operating system, in any version, with 11 megabytes of memory available on the cell phone for the free download of *‘Agente Escuta’*; using the app freely, during a period of two months, not being necessary to effectively use the app in the work routine; and fully answering the questionnaires to assess usability. For the third stage, three months of consecutive use of the app were required.

A total of 41 CHWs, out of the 91 CHWs working in the 12 selected PHCU, were invited to participate in this study to meet the determined sample number. A total of 35 CHWs participated in the study, as five CHWs did not download the app in the reference period of the study and one did not complete the user test. The distribution of CHWs in relation to location and sociodemographic characteristics is shown in Table 1.

**Table 1:** Characterization of Community Health Agents

**Cities**	**Mesoregion**	**n (%)**	**Sex**		**Average age** ± **SD (years)**	**Educational level**
			**F**	**M**		
João Câmara	Agreste Potiguar	2 (6.06%)	1	1	46.50 ± 0.70	a (n=1); d (n=1)
Santa Cruz	Agreste Potiguar	3 (9.10%)	2	1	37.34 ±8.50	b (n=2); d (n=1)
Upanema	WestPotiguar	7 (21.22%)	6	1	40.43 ± 4.71	b (n=5); d (n=1); e (n=1)
Caicó	Central Potiguar	11 (31.42%)	11	0	41.62 ± 6.20	a (n=1); b (n=6); c (n=4)
São Miguel do Gostoso	EastPotiguar	2 (6.06%)	1	1	32.50 ± 2.12	b (n=1); c (n=1)
Natal	EastPotiguar	10 (30.31%)	6	4	41.18 ± 11.37	b (n=2); c (n=7); d (n=1)
TOTAL		35 (100.00%)	27	8	40.84 ± 8.04	b (45.71%); c (34.29%)

**Caption:** F = Female; M = Male; SD = Standard deviation; a = Incomplete secondary education; b = Complete secondary education; c = Incomplete higher education; d = Complete higher education; e = Post-graduation

All participants received the downloadable file in .apk format with the prototype of the application, sent individually. Along with the file, a tutorial video on how to download the prototype on smartphones was attached.

During the study period, three CHWs, in addition to carrying out the usability test, completed three consecutive months of effective use of the app, which enabled the execution of the third stage of the study.

### Main output measures

The methodology of this study considered the guidelines of the International Organization for Standardization (ISO), a group of technical standards that establish a quality management model. To this end, the following standards were considered when choosing the evaluation tools for the *Agente Escuta*: ISO 25010 - mobile app analysis; ISO 25062 - user satisfaction and effectiveness of the tool tested; ISO 16982 - user’s opinion about the application's interface, and ISO 14998 - software evaluation.

To assess the usability of the app, the System Usability Scale (SUS) was used, one of the most accepted instruments due to its reliability^(19)^, previously translated and validated into European Portuguese^(20)^. The SUS method can be used to evaluate products, services, hardware, software, websites, applications or any other type of interface. Used in previous studies with the same purpose, it contains 10 items to be answered individually and applied anonymously.

The SUS was made available in digital online format through Google Forms, and the link was sent individually to each participant at all stages. The questionnaire was answered anonymously using the Likert Scale, in which 1 indicated complete disagreement and 5 complete agreement.

The questions were subdivided taking into account heuristics which indicate important aspects of usability ^(18)^. Thus, questions 3, 4, 7 and 10 were related to “ease of learning”, questions 5, 6 and 8 to “product efficiency”, question 2 to “ease of memorization”, question 6 to “minimization of errors” and questions 1, 4 and 9 to “satisfaction of use”.

For the analysis of SUS results, the sum of the individual contribution of each item was considered. For odd items, 1 was subtracted from the participant's response, whereas for even items, 5 was subtracted from the user's response. After obtaining the score for each item, the scores were added and the result was multiplied by 2.5. The result obtained was the participant's satisfaction index, which can vary from 0 to 100. The average and standard deviation (σ) of the satisfaction indices of all participants were calculated to obtain the app's usability level classification. The average SUS score is 68 (50th percentile) and is considered a cutoff point, that is, an average above this value suggests a good level of usability^(18,20)^.

To measure app usability and satisfaction, in addition to the SUS instrument, the Net Promoter Score (NPS)^(21)^ and an open question for observations and recommendations were used. The NPS is a simple and concise way of examining the satisfaction of patients, users or clients with a service; it is referred to as “the final question”, suggesting that it is a summary of user satisfaction of some service or product^(21)^.

The assumption is that individuals scoring 9 or 10 will give the product positive publicity; they are called ‘promoters’. Individuals answering 7 or 8 are considered indifferent (“passive”). Finally, individuals who answer 0 to 6 are likely to be dissatisfied customers and are called “detractors”. The NPS is then calculated as the difference between the percentage of “promoters” and “detractors” and can range from -100% to +100%. The higher the percentage, the greater the chance that the product will be recommended to other users^(21)^.

The NPS was made available to research participants through the same Google Forms link, but in a session after SUS, with the question “How likely are you to recommend *‘Agente Escuta’* to another user?” and the response option was made available on a Likert scale from zero to 10, where zero corresponded to “not likely” and 10 to “extremely likely”.

Regarding the third stage of the study, the participating CHWs answered an additional satisfaction questionnaire prepared based on a previous instrument^(22)^. The questionnaire contained 10 items that represented practical situations of auditory monitoring mediated by the app, with the following response options: (1) strongly disagree, (2) disagree, (3) indifferent, (4) agree, (5) strongly agree. The CHWs answered this instrument using an online form, as in the previous stages.

### Data analysis

For the descriptive analysis, the average SUS and NPS score was considered for each category of judges and for the CHWs of each municipality, in addition to the thematic categorization of the qualitative variables related to the suggestions and improvements indicated.

The inferential analysis was first performed with the Shapiro-Wilk adherence test in each group, with normal data distribution being observed in stage 1 (p= 0.078) and absence of normality in stage 2 (p= 0.047). Thus, the one-way Analysis of Variance (ANOVA) was used to compare the SUS score between the categories of judges; the Kruskal-Wallis test to compare SUS and NPS among participants in each municipality; and the Mann-Whitney test to compare usability in the perception of judges and target audience, the CHWs. The analysis was performed using the Software Statistical Package for the Social Sciences (SPSS), version 24, and the significance level adopted was 5%.

## RESULTS

**Figure 2:**
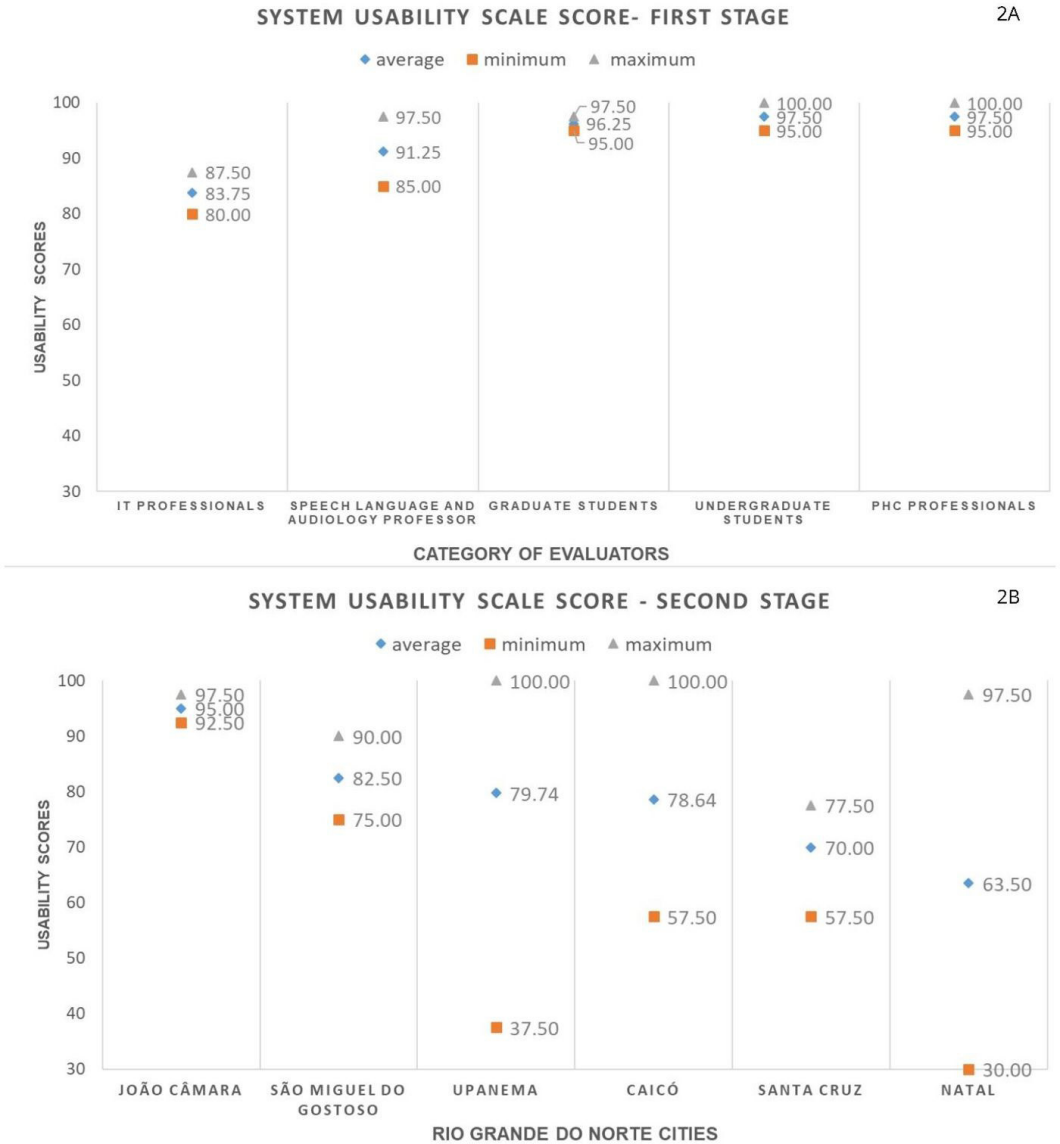
Total score average on the System Usability Scale (SUS), maximum and minimum value for each evaluator category in the first stage (2A) and for each municipality in the second stage (2B)

In the usability assessment with the SUS method in the first stage of this study, the participation of a third judge from the same category was not necessary, considering that there was no difference greater than 20 points between the evaluators (Figure 2A). All the judges evaluated the *‘Agente Escuta’* app with a score higher than the 50th percentile of the SUS, with an average of 93.50 in the total score (σ = 5.90), suggesting, according to the evaluators of the first stage, that the beta version this tool does not face major usability difficulties.

**Caption:** IT = Information Technology; PHC = Primary Health Care

It was also possible to observe that the category with the lowest usability score in the SUS was that of IT professionals (average = 83.75), and the highest scores were attributed by graduate students in speech therapy and PHC professionals (average = 97.50). The one-way ANOVA showed that there was no effect of the judges’ category on the usability score obtained [F= (4.5) = 2.593; p= 0.162], with similar evaluations.

For the analysis of the judges' intra-category agreement percentage, the responses were grouped into positive, negative or neutral. For the odd-numbered SUS items, the responses “agree” and “completely agree” were considered positive, while “disagree” and “completely disagree” were classified as negative. On the other hand, for the even items, the opposite was adopted. The percentage of agreement between judges in the same categories ranged from 80% (IT professionals), 90% (undergraduate students) and 100% for the other categories.

In the second stage, also using the SUS tool, the average scores of the 35 participating CHWs were calculated according to the municipalities (Figure 2B). The total score, calculated using the weighted average of the municipalities’ scores, was 78.21 (σ = 10.83), indicating “good” usability and above the 50th percentile of the SUS. The highest SUS average score was given by participants from the municipality of João Câmara (average = 95.00), and the lowest score was given by participants from the capital, Natal, (average = 63.50), which is also the only municipality with a score below 68 (50th percentile). The Kruskal-Wallis Test showed that the municipality of origin of the CHWs did not affect the total SUS score [X^2^ (5) = 7.637; p= 0.177].

When comparing the scores of the two stages using the Mann-Whitney test, it was clear that the perception of the judges and the target audience were different (U = 68,000; p =0.003), with a lower score for the CHWs.

**Figure 3:**
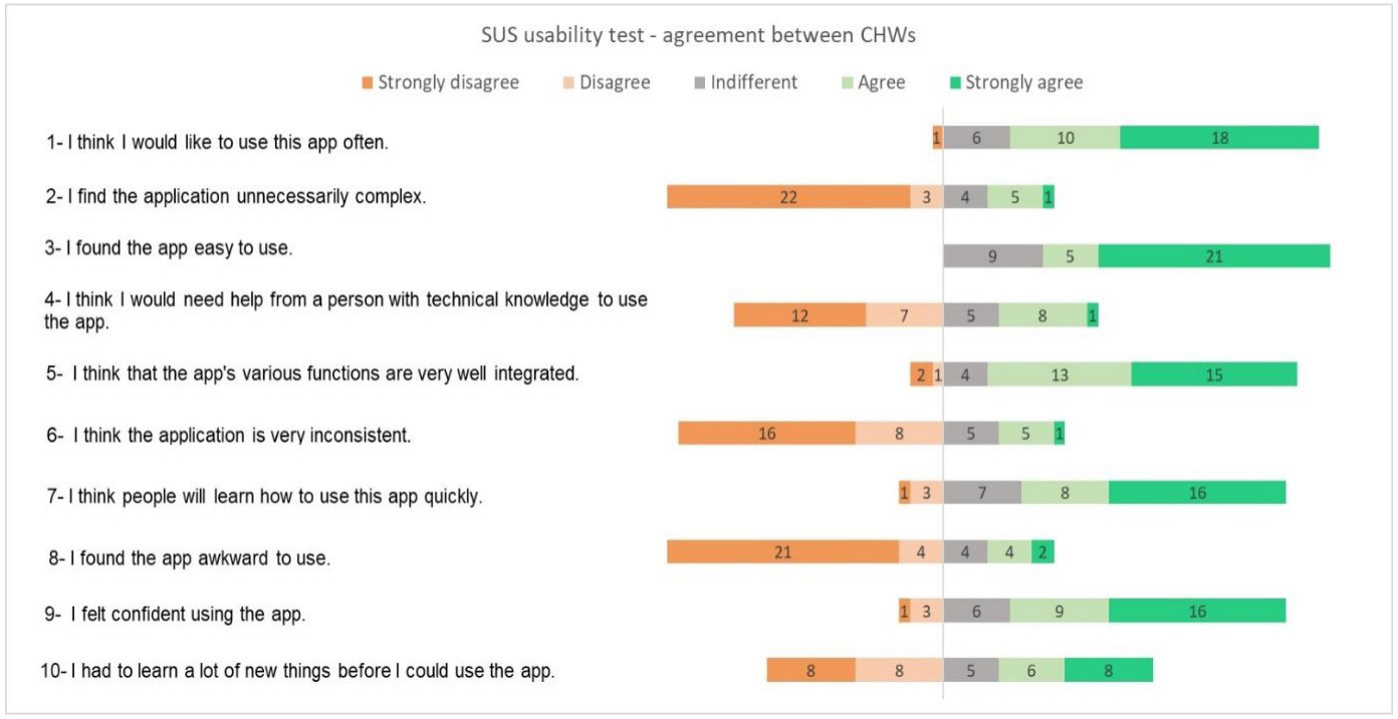
Agreement of participants' responses in the second stage on the System Usability Scale

When considering the intra-group assessment of stage 2, greater agreement was seen in relation to the second SUS statement “I find the application unnecessarily complicated” in which 22 participants (62.85%) strongly disagreed with the statement. The last statement of the questionnaire “I needed to learn several new things before I could use the application” had the greatest heterogeneity in the responses (Figure 3).

**Caption:** SUS= System Usability Scale (SUS); app = Application; CHW = Community Health Workers

Regarding heuristics^(18)^, in the judges’ assessment, a lower score was obtained for “satisfaction of use”, with an average score of 90.60. According to the evaluation of the CHWs participating in the second stage, the lowest score was for “minimizing errors”, with an average score of 73.57. Participants in both stages showed a higher score for the heuristic referring to “ease of memorization”, with an average of 97.50 in the first stage and 80.71 in the second stage.

**Figure 4:**
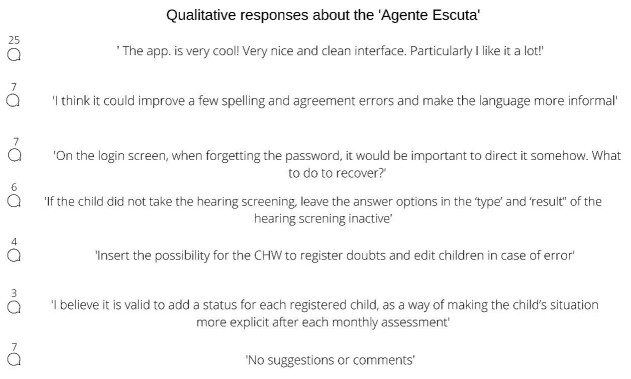
Summary of the comments and suggestions of participants in the two stages regarding the application, including number of repeated opinions

After using and evaluating the tool, participants recorded comments and suggestions to be implemented in future versions of the app. There was similarity in the responses obtained among the participants of the two stages, so they could be compiled into seven representative statements (Figure 4).

**Caption:** app = Application; CHW = Community Health Workers

**Figure 5:**
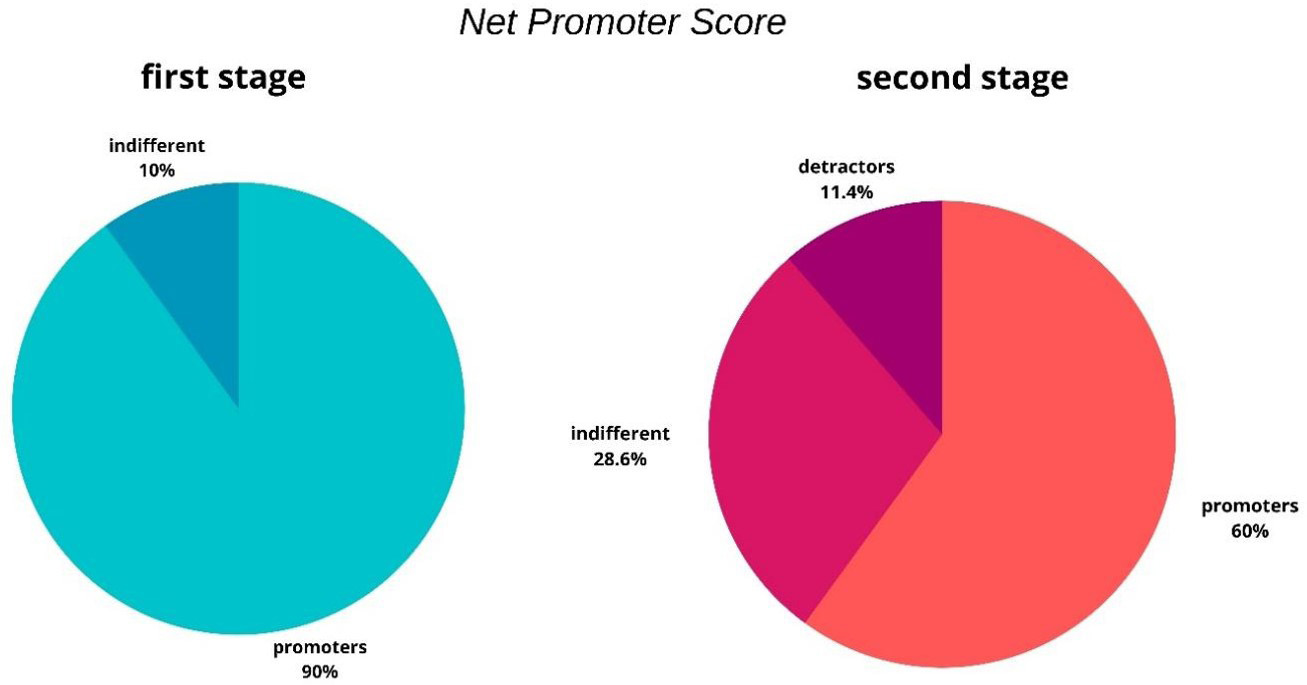
Percentage of users classified as “promoters”, “indifferent” and “detractors” in the Net Promoter Score in relation to the two stages

Regarding the final satisfaction question, asked through the NPS tool, the Mann-Whitney Test showed a difference between the judges' assessment in stage 1 and the perception of potential users in stage 2 (U= 89.500; p= 0.014). Considering the total of 10 judges, eight (80%) answered “10” on the Likert scale, one answered “9” and another answered “8”. Following the NPS criteria, 90% of the judges would be ‘promoters’, that is, they would give positive publicity to the product, and 10% would be “indifferent” (Figure 5). As the NPS calculation considers only the difference between “promoters” and “detractors”, and “detractor” users were not obtained in the first stage of the study, the NPS result was +100%.

On the other hand, in the second stage, the NPS of the 35 CHWs resulted in +48.58%. A total of 21 (60.00%) participants answered “10” and “9” on the Likert scale, 10 (28.57%) answered “8” and “7”, and four (11.42%) answered “6”, with a predominance of 'promoters' (Figure 5). The Kruskal-Wallis test showed that there was no difference in the NPS when comparing the municipalities [X^2^ (5) = 3.945; p = 0.557].

Regarding the additional satisfaction questionnaire, only three CHWs met the prerequisite of effective use of the app in their work routine for three consecutive months, and the others used it for different periods. The three participants were from different municipalities and followed up 22 children aged between one and 12 months (average of 5.55 months and standard deviation of 3.46 months). Despite the level of education of these CHWs covering incomplete secondary education, complete secondary education and incomplete higher education, the restricted number of participants does not allow any inference to be made about the impact of educational level on the results obtained. It was found that there was an agreement of answers in the items related to the importance and relevance of carrying out auditory monitoring (Questions 5, 8 and 9), positive influence of the *Escuta Game* in the revision of auditory health contents (Question 4), and desire to continue using the app in the work routine (Question 10).

There was a difference of opinion on questions related to the ease of applying the hearing and language monitoring questionnaire through the app, and to the community's acceptance of using the app as a tool (Questions 1-3, 5-7). It was found that in each topic the negative analysis was made by only one CHW, who differed in the questions.

## DISCUSSION

Among the various activities carried out by the PHC, the auditory monitoring of all children in the community during childcare consultations is recommended as one of the steps of the auditory identification and intervention program in the first years of life^(14)^.

An alternative to enhance auditory monitoring is the articulation with the CHWs, who are recognized for being the professionals most aware of the real needs of the community. To achieve this, studies around the world are investing in the development of tools that help the CHWs to monitor hearing, even in remote communities^(5,6,23,24)^.

The purpose of the app was to provide support in the structuring of a referral flow, becoming a facilitator in the implementation of hearing health actions in the CHW's work routine. The result of the usability evaluation of the ‘*Agente Escuta’*, in the first stage of this study, showed that the two categories that scored the most in the first stage are directly inserted in the reality of hearing monitoring by the PHC and in the development of research aimed at this population, that is, PHC professionals and graduate students in Speech Therapy. However, even professional IT evaluators, who are not familiar with the auditory monitoring stage in practice, kept positive evaluations.

Thus, the fact that there were no differences in the evaluations between the judges’ categories indicated that usability was well evaluated both by professionals involved in the development of technological solutions and by evaluators who experience PHC.

On the other hand, in the second stage, the general score of the CHWs was lower than in the first stage. Therefore, it is considered that the PHC professionals who participated in the first stage did not represent the CHWs in the Rio Grande do Norte municipalities, since greater difficulties were observed in the usability of the tool by the participants in the second stage.

Although apps aimed at the health area have gained prominence in recent years due to the availability of access and ease of use of functionalities that were previously only available through the computer, aspects such as internet availability, access to mobile devices aimed exclusively at work, and technical training for the handling of technological tools by health professionals is crucial for the successful implementation of m-health in the work routine^(24)^.

That said, there is a digital health initiative by the Ministry of Health, “*Informatiza APS*”, which aims to support the computerization of health units and the qualification of PHC data throughout the country^(7)^. Some apps for smartphones have already been developed to help PHC professionals in their work routines, namely: e-SUS AD, e-SUS territory and e-SUS AB. However, the analysis of usability or user satisfaction regarding these technological solutions was not found in the researched literature.

In Brazilian studies on remote hearing health training through CD-ROM, Cybertutor and online course available on the platform of the Ministry of Health, it was found that CHW professionals had difficulties with basic information technology. These data showed that adherence to permanent health education strategies may have been impacted by the technical difficulty in handling web and desktop interface programs^(6,25)^. It was thought that the CHW's difficulties with basic computing were linked to poor access to computers in the PHC.

A survey carried out in 2020 pointed out that the number of smartphones in Brazil is equivalent to more than one device per inhabitant, with a total of 234 million^(26)^. These data about the popularization of smartphones raised the hypothesis that mobile applications aimed at auditory monitoring could have an advantage over strategies with web and desktop implementation. However, it was observed in this study that, even with wide access to smartphones, CHW professionals have basic technical difficulties in mobile applications, such as finding the application on the home screen of the mobile device, registering a password and remembering the email.

In this way, mobile applicability tools aimed at this user audience need a well-designed interface so that users can extract as much as possible from the app, without major technical difficulties. Dissatisfaction in use can cause a bad impression on the users and they may give up using the app, which is not desirable^(20)^.

Usability is linked to the quality of user interaction with the interface, be it a system, tool or mobile application. Usability evaluations seek to analyze the system interface quality, and whether that product is intuitive enough to the point of not having so many or almost no failures that affect the use, with a quality that is at least acceptable. Usability tests aim to find usability issues in interfaces according to how users use them. In this case, users test the system functionalities, reporting possible interaction issues during use^(20)^.

In Bangladesh, a study carried out a review and assessed the usability of the different m-Health apps in the country^(27)^. The results pointed to serious usability issues identified by the SUS method. Participating evaluators indicated that most health-oriented mobile applications developed in Bangladesh scored lower on the aesthetic and graphic design heuristic. The authors concluded that the unintuitive design could explain the lack of adherence to the use of apps in the country's health services.

In the results of this study, the graphic design and aesthetics of the app were well evaluated in both the first and second stages. In addition, in the judges' perception, a higher score was obtained for questions related to “ease of learning”, with an average score of 97.50, which may be linked to the well-structured and easy-to-use graphical interface of the app.

In the second stage, the participating CHWs evaluated the interface in a similar way, but with an average score of 80.71. When considering the score of questions related to the interface and the low percentage of CHWs who used the application in their work routines (8.57%), it was possible to observe that although the app graphic design has a good score, this variable did not directly reflect on adherence to the effective use of the app. Therefore, the actual implementation of the application was not linked to graphic design satisfaction, as seen in the systematic review in Bangladesh^(27)^.

The lowest score among the SUS questions, in the judges’ evaluation of the first stage of the study, was in “satisfaction of use”, with an average score of 89.00. Although it was the lowest score, it still represents an excellent usability value^(20)^. In the second stage, the lowest score was for “ease of learning”, with 71.00. It is believed that this drop in scores related to satisfaction and ease of learning can be explained by the fact that some of the app features did not meet the evaluators’ expectations such as, for example, the absence of the “I forgot the password” option and the “doubts” tab, which does not offer the possibility for the user to register their doubts directly in the app, and the TAN response options during the registration of children monitored by the app, which end up leaving the possibility of filling in the wrong data.

Filling errors in children's registration can be minimized by the ADM version of the app, which has access to all information registered by users. Therefore, if there is any incompatible information in the data regarding the TAN, the ADM has the possibility of correcting them through its web version.

In addition, the version of this app was designed with the “doubts” tab in a forum format, as suggested by the evaluators. However, during the development of the interface, it was observed that the option of inserting questions directly into the app would make the software more robust and, consequently, would require more free memory on the device for the download. Considering the possibility of users not being able to download the app due to lack of available memory on their smartphones, it was decided to leave the “doubts” tab with the questions fixed and editable only by the ADM, who was in direct contact with the participants' doubts.

Other aspects highlighted in the evaluators' responses were typing errors and suggestions for inserting simple features, such as the “I forgot the password” option right on the login screen. These topics are easy to correct and improvements can be implemented in future versions of the app.

Regarding the NPS evaluation result, a study conducted in South Africa with a hybrid application (web and face-to-face) to monitor the hearing of patients resulted in a score of +87% in the users' NPS. The authors pointed out that, given this NPS value, it is highly likely that users will recommend the hybrid clinic to friends and family^(28)^.

Considering the results of the NPS assessment in this study, +100% of the evaluators in the first stage would probably recommend the use of the app to other users, remaining in the category of ‘promoters’ as regards NPS assessment. As for the participants of the second stage, who scored +48.58% in the NPS, they demonstrate that, although the tool does not present serious usability issues, it would still not be as recommended for other users when compared to the participants in the first stage.

A Brazilian study on hearing health training showed that professionals who dropped out of the study pointed out as main reasons the turnover of professionals and positions, lack of participation of managers, and high demand for CHW-related activities^(4)^. The lack of clarity about the CHW's attributions can cause overload in the workday and because they do not have a clear career plan these professionals end up performing several non-standard tasks. This is a relevant finding for the implementation of new tools, as in addition to adequate usability, the motivation of professionals for effective use is of critical importance.

Satisfaction with app use in the work routine was impaired, since, during the period of this study, the functions of the CHWs were redirected to support actions by health professionals focused on the COVID-19 pandemic, such as intense testing and the national vaccination campaign. Thus, despite the promising results, the small number of CHWs who used the app effectively in their work routine in the third stage does not allow generalizing the data obtained, which is a limitation of the study.

## CONCLUSION

The smartphone app prototype ‘*Agente Escuta*’, developed to help the auditory and language monitoring stage in the PHC, presented good usability according to participants in Rio Grande do Norte, with 90% of the judges and 60% of the CHWs giving positive publicity to the product. The CHWs who used the app in their routine agreed with the importance and relevance of carrying out auditory monitoring, indicated the positive influence of the *Escuta* Game in reviewing content on hearing health and showed interest in continuing to use the app in their work routine.

Improvements should be implemented in the next versions of the application or in proposing other technological solutions for the target audience, according to the suggestions of the evaluators of this study.
