# Supplementary material for: Usability evaluation of the Agente Escuta application: translational research
Source: Codas. 2023 Sep 15;35(4):e20220149. doi: 10.1590/2317-1782/20232022149en (PMC10546923; doi:10.1590/2317-1782/20232022149en)
Supplement: Translation

# Translation: Avaliação da usabilidade do aplicativo *Agente Escuta*: uma pesquisa translacional

**DOI:** 10.1590/2317-1782/20232022149pt

**Authors:** Alice Andrade Lopes Amorim, Kátia de Freitas Alvarenga, Lilian Cássia Bórnia Jacob, Eliene Silva Araújo

**Affiliations:** 1 Programa Associado de Pós-graduação em Fonoaudiologia, Universidade Federal do Rio Grande do Norte - UFRN - Natal (RN), Brasil.; 2 Faculdade de Odontologia de Bauru - FOB, Universidade de São Paulo - USP - Bauru (SP), Brasil.

**Keywords:** Design Centrado no Usuário, Educação em Saúde, Saúde Móvel, Agentes Comunitários de Saúde, Política Nacional de Atenção à Saúde Auditiva

## Abstract

**Objetivo:**

Avaliar a usabilidade e satisfação dos usuários com a interface do aplicativo “Agente Escuta”, além de identificar problemas e possibilidades de melhorias.

**Método:**

Estudo translacional exploratório descritivo, caracterizado por um teste de usabilidade com abordagem quanti-qualitativa, subdividido em três etapas: (I) avaliação prévia da usabilidade por 10 juízes, incluindo estudantes, profissionais da atenção primária, docentes e pesquisadores em Tecnologia da Informação e Fonoaudiologia; (II) avaliação do aplicativo pelo público-alvo, agentes comunitários de saúde de seis municípios potiguares; (III) Avaliação da satisfação dos agentes que utilizaram o aplicativo na rotina de trabalho. Utilizou-se o *System Usability Scale* e pelo *Net Promoter Score*, além de uma avaliação qualitativa das opiniões.

**Resultados:**

A usabilidade foi avaliada como excelente pelos juízes, independentemente da categoria. Na avaliação pelos agentes comunitários de saúde, a usabilidade foi considerada boa e não houve efeito do município de origem. Constatou-se que a percepção dos juízes e do público-alvo foram distintas, com menor escore para os participantes da segunda etapa, no entanto, a maioria dariam publicidade positiva ao produto. A heurística com maior pontuação foi a facilidade de memorização e os participantes da terceira etapa mostraram-se interessados em continuar utilizando a ferramenta na prática, mesmo após o término do estudo.

**Conclusão:**

O protótipo do Agente Escuta apresentou boa usabilidade e satisfação e foram identificados aspectos que poderão ser aprimorados em soluções futuras.

## INTRODUÇÃO

A expansão e a qualificação da Atenção Primária à Saúde (APS) têm sido vistas como principais iniciativas para produzir mudanças qualitativas na saúde para as próximas décadas em todo o mundo^(1)^. No Brasil, a Política Nacional de Atenção Básica (PNAB) prevê a articulação de instituições em parceria com as secretarias de saúde a fim de propiciar a educação permanente e continuada para os profissionais da APS^(2)^.

Ao considerar especificamente a saúde auditiva, o relatório da Organização Mundial da Saúde (OMS) em 2021 destaca projeções alarmantes em relação à prevalência da perda auditiva, com estimativa de que 900 milhões de pessoas poderão ter algum grau de perda auditiva até 2050. Além disso, reitera o quantitativo de causas passíveis de prevenção, bem como o custo global anual de US$ 750 bilhões para a perda auditiva não tratada^(3)^. Assim, é inegável a importância da APS na área de saúde auditiva, bem como a necessidade de desenvolvimento de ferramentas com alcance às diferentes localidades do país.

Diante deste cenário, nota-se um crescimento considerável de programas e tecnologias inovadoras que visam fortalecer este nível de atenção. Soluções tecnológicas em telessaúde começaram a ser desenvolvidas para os profissionais da saúde, como os Agente Comunitários de Saúde (ACS), no intuito de superar as barreiras ao acesso em cuidados com a saúde auditiva, usando *smartphones*, tablets, computadores e outros dispositivos portáteis^(4)^. Assim, inovações na área da educação em saúde baseadas em *mHealth* (saúde móvel) já são realidade e cresceram exponencialmente nos últimos cinco anos em outros países^(5,6)^.

No Brasil, a tendência não tem sido diferente, atualmente têm-se o programa “Informatiza APS” que faz parte da estratégia de saúde digital do Ministério da Saúde^(7)^ e os apps e-SUS AD^(8)^ e e-SUS território^(9)^, com o intuito de facilitar o processo de trabalho dos ACS locais.

Na África do Sul, desde os anos 2000, são utilizadas tecnologias de saúde móvel no programa de saúde auditiva^(10)^. Essas tecnologias de *mHealth* validadas, permitiram a realização de triagens auditivas pela APS, usando testes automatizados com a interface de *smartphone.* Diante disso, os ACS começaram a realizar a triagem auditiva para crianças em uma variedade de contextos comunitários, incluindo visitas domiciliares e o acompanhamento do desenvolvimento da primeira infância^(11)^.

Avaliações desse modelo demonstraram que os ACS podem ser treinados para rastrear crianças de maneira confiável e com eficiência de tempo. Porém, há desafios neste programa, como os níveis de ruído ambiental durante os exames, que devem estar adequados para que não influenciem nas taxas de referência da triagem auditiva. Um outro desafio está em monitorar a qualidade da triagem auditiva feita pelos ACS^(11)^.

Nesse sentido, a avaliação da usabilidade de aplicações móveis representa uma estratégia de assegurar que os sistemas interativos sejam adaptados aos usuários, às suas tarefas e que não haja quocientes negativos de seu uso. O objetivo de uma avaliação de usabilidade é averiguar o grau em que um sistema ou produto é eficaz, ou seja, quão bem o desempenho do sistema atende às tarefas para as quais foi projetado; eficiente, isto é, quantos recursos, como tempo ou esforço, são necessários para usar o sistema a fim de realizar tarefas para as quais o sistema foi projetado, e por fim, se o mesmo favorece atitudes e respostas positivas dos pretensos usuários^(12)^.

Posto isso, o aplicativo “Agente Escuta”, desenvolvido neste estudo, foi idealizado com o propósito de auxiliar os profissionais da APS no acompanhamento do desenvolvimento da audição e da linguagem, com base nos marcos do desenvolvimento. Um outro objetivo do app é o de promover a educação continuada em saúde auditiva dos ACS de forma interativa.

Porém, por se tratar de um app inédito, não existem estudos prévios sobre sua usabilidade ou a caracterização da sua implementação em contexto brasileiro. Diante disso, o objetivo do presente estudo foi verificar a usabilidade e satisfação dos usuários com a interface do aplicativo “Agente Escuta”, além de identificar problemas e possibilidades de melhorias.

## MÉTODO

Trata-se de um estudo translacional exploratório com delineamento prospectivo descritivo. Foi realizado por meio de um teste de usabilidade do app “Agente Escuta” com abordagem quantitativa e qualitativa, a fim de identificar dificuldades e possíveis melhorias na ferramenta.

O estudo foi estruturado em três etapas, a saber: (I) avaliação prévia da usabilidade por 10 juízes, incluindo estudantes, profissionais da atenção primária, docentes e pesquisadores em Tecnologia da Informação e Fonoaudiologia; (II) avaliação do aplicativo pelo público-alvo, ACS de seis municípios do Rio Grande do Norte (RN), Brasil; (III) Avaliação da satisfação dos ACS que utilizaram o aplicativo na rotina de trabalho.

Seguindo as orientações das Diretrizes e Normas Regulamentadoras de Pesquisa Envolvendo Seres Humanos (Resolução 466/12), o estudo só foi iniciado após aprovação do Comitê de Ética em Pesquisa da Instituição (parecer número 4.695.580) e mediante assinatura do Termo de Consentimento Livre e Esclarecido, sendo fornecido todo esclarecimento necessário a respeito da sua participação no estudo.

### Contexto

Em parceria com o Instituto Metrópole Digital da Universidade Federal do Rio Grande do Norte (IMD/UFRN), reconhecido por seus projetos com interface na saúde, o protótipo do aplicativo foi desenvolvido no período de um ano, entre 2020 e 2021. A primeira versão da ferramenta foi desenvolvida com uma média de 15.000 linhas de código e em linguagem predominantemente *Javascript,* sendo programada a *back-end* e *front-end* da versão móvel e a *dashboard* da versão *web*. O registro do software no Instituto Nacional da Propriedade Industrial (INPI) foi expedido em novembro/2021 sob número de protocolo BR512021002590-3.

O app funciona sob dois eixos principais, o da educação continuada em saúde auditiva e o do acompanhamento do desenvolvimento da audição e da linguagem das crianças de zero a 12 meses de idade. No eixo da educação continuada, são disponibilizadas abas para revisão de conteúdos, a saber: as dúvidas mais frequentes sobre saúde auditiva levantadas pelos ACS durante o uso do app, um fluxograma com os marcos de desenvolvimento nos dois primeiros anos de vida e o Escuta Game, apresentado na tela inicial, que contém situações diárias sobre saúde auditiva.

Para a construção de suas funcionalidades, foi realizado um levantamento de ferramentas já validadas, como cartilhas de orientação baseadas nos marcos do desenvolvimento, questionários e diretrizes internacionais e nacionais^(13-17)^. A Figura 1 sintetiza as funcionalidades do app.

**Figura 1:** Infográfico síntese das funcionalidades do “Agente Escuta” e tela representativa do questionário de acompanhamento, com mensagem indicativa de falha

**Legenda:** ACS = Agentes Comunitários de Saúde

No eixo do acompanhamento da audição e da linguagem, o aplicativo disponibiliza as questões referentes à idade da criança cadastrada^(17)^. Ao respondê-las, o questionário específico de cada criança só ficará novamente disponível no mês seguinte, com a faixa etária subsequente, o que diminui as chances de preenchimento duplo no mês. De acordo com o critério “falha” proposto no questionário de acompanhamento^(17)^, o app automaticamente referencia o usuário para a aba “centros de saúde” onde estão elencados todos os centros de saúde auditiva da região a fim de facilitar o encaminhamento para avaliação audiológica.

Todas estas informações são enviadas para o banco de dados MySQL, que consiste em um banco de dados relacional, conectado a um servidor privado com tecnologia baseada em criptografia e que podem ser acessadas continuamente apenas pelo perfil de administrador (ADM), que no caso são os pesquisadores responsáveis.

Na versão do ADM são disponibilizadas informações sobre o quantitativo de questionários aplicados, acertos e erros no jogo, os resultados de cada criança acompanhada, localidade, IRDA das crianças cadastradas, se realizou a TAN e os resultados obtidos.

### Participantes

Para a avaliação prévia da usabilidade do app foram convidados dois juízes de cada categoria, não envolvidos com o desenvolvimento do app ou em outras etapas da pesquisa, a saber: estudantes de graduação e pós-graduação em Fonoaudiologia, ACS, docentes e pesquisadores em Tecnologia da Informação e em Fonoaudiologia. Os 10 avaliadores foram selecionados por sua atuação profissional, histórico de desenvolvimento de aplicações móveis com interface na saúde e/ou participação em estágio universitário com ações diretamente ligadas à promoção da saúde auditiva infantil na APS.

Optou-se pela inclusão de dois participantes em cada categoria, a fim de se obter uma análise pareada. Um terceiro juiz seria convidado caso os escores totais da escala de usabilidade entre os dois avaliadores apresentassem mais do que 20 pontos de diferença na avaliação individual. Ao total, participaram desta primeira etapa, três juízes do sexo masculino e sete do sexo feminino, com idades entre 21 e 48 anos.

Na segunda etapa da pesquisa a usabilidade do app foi avaliada por ACS interessados em utilizar a ferramenta em sua prática diária. A definição do n amostral mínimo, teve como referencial a estimativa de que um único usuário é capaz de encontrar em média 31% dos problemas de usabilidade, cinco usuários são suficientes para identificar 85% dos problemas e que ao realizar o teste com 15 usuários, aproximadamente, 100% dos problemas podem ser identificados^(18)^. Assim, definiu-se o tamanho da amostra de 15 ACS, com representatividade mínima de cinco usuários em cada mesorregião do RN.

A fim de garantir maior representatividade do contexto geral do RN, optou-se por selecionar os municípios mediante sorteio por conglomerados. Para o sorteio considerou-se as quatro mesorregiões do RN, a saber: Agreste Potiguar, Central Potiguar, Leste Potiguar e Oeste Potiguar. Com exceção de Natal que foi selecionada por ser a capital do estado, os municípios de cada mesorregião foram sorteados, sendo eles: Caicó, Upanema, Santa Cruz, São Miguel do Gostoso e João Câmara.

Em comum acordo com os secretários municipais de saúde, para cada município sorteado foram selecionadas UBS inseridas em bairros com maior quantitativo de crianças com idades entre zero e 12 meses.

Como critérios de inclusão para as duas primeiras etapas adotou-se possuir *smartphone* com sistema operacional Android, em qualquer versão, com 11 megabytes de memória disponível no celular para o download gratuito do “Agente Escuta”; utilizar o app de forma livre, durante o período de dois meses, sem a necessidade de utilização efetiva do app na rotina de trabalho, além de responder de forma completa os questionários para avaliação da usabilidade. Para a terceira etapa exigiu-se três meses de uso consecutivo do app.

Do total de 91 ACS atuantes nas 12 UBS selecionadas, 41 ACS foram convidados a participar do presente estudo com vistas a atender ao n amostral determinado. Participaram do estudo 35 ACS, pois cinco ACS não fizeram download do app no período de referência do estudo e um não realizou o teste de usuário de forma completa. A distribuição dos ACS em relação à localidade e as características sociodemográficas está apresentada na Tabela 1.

**Tabela 1:** Caracterização dos Agentes Comunitários de Saúde participantes

**Município**	**Mesorregião**	**n (%)**	**Sexo**		**Idade média** ± **dP (anos)**	**Nível instrucional**
			**F**	**M**		
João Câmara	Agreste Potiguar	2 (6,06%)	1	1	46,50 ± 0,70	a (n=1); d (n=1)
Santa Cruz	Agreste Potiguar	3 (9,10%)	2	1	37,34 ±8,50	b (n=2); d (n=1)
Upanema	Oeste Potiguar	7 (21,22%)	6	1	40,43 ± 4,71	b (n=5); d (n=1); e (n=1)
Caicó	Central Potiguar	11 (31,42%)	11	0	41,62 ± 6,20	a (n=1); b (n=6); c (n=4)
São Miguel do Gostoso	Leste Potiguar	2 (6,06%)	1	1	32,50 ± 2,12	b (n=1); c (n=1)
Natal	Leste Potiguar	10 (30,31%)	6	4	41,18 ± 11,37	b (n=2); c (n=7); d (n=1)
TOTAL		35 (100,00%)	27	8	40,84 ± 8,04	b (45,71%); c (34,29%)

**Legenda:** F = Feminino; M = Masculino; dP = Desvio padrão; a = Ensino médio incompleto; b = Ensino médio completo; c = Ensino superior incompleto; d = Ensino superior completo; e = Pós-graduação

Todos os participantes receberam o arquivo para download em formato *.apk* com o protótipo do aplicativo, enviado de forma individual. Juntamente com o arquivo, foi anexado um tutorial em vídeo de como baixar o protótipo em seus *smartphones*.

No período do estudo, três ACS além de realizarem o teste de usabilidade, completaram três meses consecutivos de uso efetivo do app, o que viabilizou a execução da terceira etapa do estudo.

### Medidas de saída principais

A metodologia deste estudo considerou as diretrizes da Organização Internacional de Normalização (ISO), um grupo de normas técnicas que estabelece um modelo de gestão da qualidade. Para tanto, foram consideradas na escolha das ferramentas de avaliação do Agente Escuta as normas: ISO 25010- referente a análise de app móveis; ISO 25062- que diz respeito a satisfação do usuário e eficácia da ferramenta testada; ISO 16982- opinião dos usuários sobre a interface do aplicativo e ISO 14998 - avaliação do produto software.

Para avaliar a usabilidade do app foi utilizada a *System Usability Scale* (SUS), um dos instrumentos mais aceitos devido à sua confiabilidade^(19)^ e previamente traduzido e validado para o português europeu^(20)^. O método SUS pode ser utilizado para avaliar produtos, serviços, *hardware*, *software, websites,* aplicações ou qualquer outro tipo de interface. Utilizado em estudos prévios, com a mesma finalidade, contém 10 itens a serem respondidos individualmente e aplicados de forma anônima.

O SUS foi disponibilizado em formato digital on-line pelo *Google Forms*, cujo link foi enviado individualmente para cada participante em todas as etapas. O questionário foi respondido de forma anônima, por meio da Escala de Likert, em que 1 foi indicativo de forte desacordo e 5 de forte concordância.

As questões foram subdivididas levando em consideração heurísticas que indicam aspectos importantes sobre a usabilidade^(18)^. Sendo assim, as perguntas 3, 4, 7 e 10 relacionadas a “facilidade de aprendizagem”, as questões 5, 6 e 8 associadas à “eficiência do produto”, a questão 2 relacionada a “facilidade de memorização”, questão 6 à “minimização de erros” e perguntas 1, 4 e 9 sobre a “satisfação do uso”.

Para a análise dos resultados do SUS considerou-se a soma da contribuição individual de cada item. Para os itens ímpares foi subtraído 1 à resposta do participante, ao passo que para os itens pares foi subtraído 5 a resposta do usuário. Depois de obter o escore de cada item, somou-se os escores e multiplicou-se o resultado por 2,5. O resultado obtido foi o índice de satisfação do participante que pode variar de 0 a 100. Foi realizada a média e desvio padrão (σ) dos índices de satisfação de todos os participantes para obter-se a classificação do nível de usabilidade do app. A pontuação média do SUS é 68 (percentil 50) e se configura como um ponto de corte, ou seja, uma média acima desse valor sugere um bom nível de usabilidade^(18,20)^.

Para a mensuração da usabilidade e satisfação do app, além do instrumento SUS, foi utilizado o *Net Promoter Score* (NPS)^(21)^ e uma questão aberta para observações e recomendações. O NPS representa uma forma simples e concisa de examinar a satisfação dos pacientes, usuários ou clientes diante de um serviço; é referido como “a questão final”, sugerindo que é um resumo da satisfação do usuário de algum serviço ou produto^(21)^.

A suposição é que os indivíduos com pontuação 9 ou 10 darão publicidade positiva ao produto; eles são chamados de 'promotores'. Indivíduos que respondem 7 ou 8 são considerados indiferentes (“passivos”). Por fim, os indivíduos que respondem de 0 a 6 provavelmente são clientes insatisfeitos e são rotulados como “detratores”. O NPS é então calculado como a diferença entre a porcentagem de “promotores” e a de “detratores”, e pode variar de -100% a +100%. Quanto maior a porcentagem mais o produto tenderá a ser indicado a outros usuários^(21)^.

O NPS foi disponibilizado aos participantes da pesquisa no mesmo link do *Google Forms*, porém em uma sessão após o SUS, com a pergunta “Qual a probabilidade de você recomendar o ‘Agente Escuta’ a um outro usuário?” e foi disponibilizada a opção de resposta em uma escala Likert de zero a 10, onde o zero estava correspondente a “nada provável” e o 10 a “extremamente provável”.

Ao considerar a terceira etapa do estudo, os ACS participantes responderam um questionário adicional de satisfação, elaborado com base em um instrumento prévio^(22)^. O questionário contém 10 itens que representam situações práticas do acompanhamento auditivo mediado pelo app, com as categorias de resposta: (1) discordo totalmente, (2) discordo, (3) neutro, (4) concordo, (5) concordo totalmente. De forma similar às etapas anteriores, os ACS responderam este instrumento por meio de formulário online.

### Análise dos dados

Para a análise descritiva, foi considerado o escore médio do SUS e NPS para cada categoria de juízes e para os ACS de cada município, além da categorização temática das variáveis qualitativas relacionadas às sugestões e melhorias indicadas.

A análise inferencial foi realizada primeiramente com o teste de aderência Shapiro-Wilk em cada grupo, sendo constatada distribuição normal dos dados da etapa 1 (p= 0,078) e ausência de normalidade na etapa 2 (p= 0,047). Assim, empregou-se a Análise de Variância (ANOVA) de uma via para comparar o escore do SUS entre as categorias de juízes; o teste de Kruskal-Wallis para a comparação do SUS e do NPS entre os participantes de cada município; e o teste de Mann-Whitney para comparar a usabilidade na percepção dos juízes e do público-alvo, os ACS. A análise foi realizada por meio do *Software Statistical Package for the Social Sciences* (SPSS), versão 24 e o nível de significância adotado foi 5%.

## RESULTADOS

Na avaliação da usabilidade pelo método SUS na primeira etapa deste estudo, não foi necessária a participação de um terceiro juiz da mesma categoria, tendo em vista que não houve diferença superior a 20 pontos entre os avaliadores (Figura 2A). Nota-se que todos os juízes avaliaram o app “Agente Escuta” com uma pontuação superior ao percentil 50 do SUS, com média de 93,50 no escore total (σ = 5,90), sugerindo, segundo os avaliadores da primeira etapa, que a versão beta desta ferramenta não enfrenta grandes dificuldades na usabilidade.

**Figura 2:** Média do escore total no *System Usability Scale* (SUS), valor máximo e valor mínimo para cada categoria de avaliador na primeira etapa (2A) e para cada município na segunda etapa (2B)

**Legenda:** TI = Tecnologia da Informação; APS = Atenção Primária à Saúde.

Além disso, foi possível observar que a categoria com menor escore de usabilidade no SUS, foi a de profissionais de T.I (média = 83,75) e as maiores pontuações foram atribuídas pelos alunos de pós-graduação em fonoaudiologia e profissionais da APS (média = 97,50). A ANOVA a um fator evidenciou que não existiu efeito da categoria dos juízes sobre o escore de usabilidade obtido [F= (4, 5) = 2,593; p= 0,162], com avaliações similares.

Para análise do percentual de concordância intra-categoria dos juízes, as respostas foram agrupadas entre positivas, negativas ou neutras. Para os itens ímpares do SUS, as respostas “concordo” e “concordo plenamente” foram consideradas positivas, enquanto “discordo” e “discordo plenamente” foram classificadas como negativas. Por outro lado, para os itens pares, adotou-se o oposto. O percentual de concordância entre os juízes das mesmas categorias variou entre 80% (profissionais da T.I), 90% (alunos de graduação) e 100% para as demais categorias.

Na segunda etapa, também por meio da ferramenta SUS, foi realizada a média dos escores dos 35 ACS participantes, de acordo com os municípios (Figura 2B). O escore total, calculado com a média ponderada entre os escores dos municípios, foi de 78,21 (σ = 10,83), apontando uma usabilidade “boa” e acima do percentil 50 do SUS. A maior pontuação média do SUS foi dos participantes do município de João Câmara (média = 95,00) e o menor escore dos participantes da capital Natal (média = 63,50), sendo também o único município com o escore abaixo de 68 (percentil 50). Constatou-se pelo Teste de Kruskal-Wallis que não houve efeito do município de origem dos ACS no escore total do SUS [X^2^ (5) = 7,637; p= 0,177].

Na comparação dos escore das duas fases por meio do teste de Mann-Whitney, foi notória que a percepção dos juízes e do público-alvo foram distintas (U = 68,000; p =0,003), com menor escore para os ACS.

Ao considerar a avaliação intra-grupo da etapa 2, foi visto maior concordância em relação à segunda afirmação do SUS “eu acho o aplicativo desnecessariamente complicado” em que 22 participantes (62,85%) discordaram fortemente da afirmativa. Já a última afirmação do questionário “Eu precisei aprender várias coisas novas antes de conseguir usar o aplicativo” foi a que obteve maior heterogeneidade nas respostas (Figura 3).

**Figura 3:** Concordância das respostas dos participantes da segunda etapa no *System Usability Scale*

**Legenda:** SUS= *System Usability Scale*; app = aplicativo; ACS = Agentes Comunitários de Saúde

No que concerne as heurísticas^(18)^, na avaliação pelos juízes, obteve-se menor pontuação em “satisfação do uso”, com escore médio de 90,60. Já de acordo com a avaliação dos ACS participantes da segunda etapa o menor escore foi em relação a “minimização de erros”, com pontuação média de 73,57. Os participantes, de ambas as etapas, apontaram um maior escore para a heurística referente a “facilidade de memorização”, com média de 97,50 na primeira fase e 80,71 na segunda.

Após o uso e a avaliação da ferramenta, os participantes registraram comentários e sugestões para implementação em futuras versões do app. Houve similaridade nas respostas obtidas entre os participantes das duas etapas, assim as mesmas puderam ser compiladas em sete falas representativas (Figura 4).

**Figura 4:** Síntese dos comentários e sugestões dos participantes das duas etapas em relação ao aplicativo avaliado, com o quantitativo de recorrência das opiniões

**Legenda:** app = aplicativo; ACS = Agentes Comunitários de Saúde

Já referente à pergunta de satisfação final, realizada por meio da ferramenta NPS, o Teste de Mann-Whitney mostrou diferença entre a avaliação dos juízes na etapa 1 e a percepção dos usuários em potencial, participantes da etapa 2 (U= 89,500; p= 0,014). Do total de 10 juízes, oito (80%) responderam “10” na escala Likert, um avaliador respondeu “9” e outro avaliador respondeu “8”. Seguindo os critérios do NPS, 90% dos juízes seriam ‘promotores’, ou seja, dariam publicidade positiva ao produto e 10% seria “indiferente” (Figura 5). Como o cálculo do NPS considera apenas a diferença entre os “promotores” e “detratores” e não foram obtidos usuários “detratores” na primeira etapa do estudo, o resultado no NPS foi de +100%.

**Figura 5:** Percentual de usuários classificados como “promotores”, “indiferentes” e “detratores” no *Net Promoter Score* em relação às duas etapas

Por outro lado, na segunda etapa, o NPS referente aos 35 ACS resultou em +48,58%. Desse total, 21 (60,00%) responderam entre “10” e “9” na escala Likert, 10 (28,57%) responderam “8” e “7” e quatro (11,42%) responderam “6”, com predomínio de ‘promotores’ (Figura 5). O teste de Kruskal-Wallis mostrou que não houve diferença no NPS ao comparar os municípios [X^2^ (5) = 3,945 ; p = 0,557].

Em relação ao questionário adicional de satisfação, apenas três ACS atenderam ao pré-requisito de uso efetivo do app em sua rotina de trabalho por três meses consecutivos, sendo que os demais utilizaram por um período variado. Os três participantes eram de municípios distintos e acompanharam o total de 22 crianças, na faixa etária de um a 12 meses (média de 5,55 meses e desvio-padrão de 3,46 meses). Apesar do nível de escolaridade destes ACS abranger ensino médio incompleto, médio completo e superior incompleto, o número restrito de participantes não permite fazer qualquer inferência do impacto do nível instrucional nos resultados obtidos. Constatou-se que houve concordância de respostas nos quesitos relacionados à importância e relevância de se realizar o acompanhamento auditivo (Questões 5, 8 e 9), sobre a influência positiva do Escuta Game na revisão dos conteúdos sobre saúde auditiva (Questão 4) e sobre o desejo de continuar utilizando o app na rotina de trabalho (Questão 10).

A divergência de opinião foi observada nos quesitos relacionados à facilidade de aplicação do questionário de acompanhamento da audição e da linguagem por meio do app, assim como a aceitação da comunidade do uso do aplicativo como ferramenta (questões 1-3, 5-7). Constatou-se que em cada tópico a análise negativa foi realizada por apenas um ACS, que diferiu entre as questões.

## DISCUSSÃO

Dentre as diversas atividades desempenhadas pela APS, o acompanhamento auditivo de todas as crianças da comunidade durante as consultas de puericultura é preconizado como uma das etapas do programa de identificação e intervenção auditiva nos primeiros anos de vida^(14)^.

Uma alternativa para potencializar o acompanhamento auditivo é a articulação com os ACS, que são reconhecidos por serem os profissionais mais cientes das reais necessidades da comunidade. Para isso, estudos ao redor do mundo estão investindo no desenvolvimento de ferramentas que auxiliem o ACS no acompanhamento da audição, mesmo em comunidades remotas^(5,6,23,24)^.

O intuito do app foi de fornecer apoio na estruturação de um fluxo de encaminhamento, tornando-se um facilitador na implementação de ações de saúde auditiva na rotina de trabalho dos ACS. O resultado da avaliação da usabilidade do Agente Escuta, na primeira etapa deste estudo, revelou que as duas categorias que mais pontuaram na primeira fase, estão inseridas diretamente na realidade do acompanhamento auditivo pela APS e no desenvolvimento de pesquisas voltadas para essa população, ou seja, os profissionais da APS e os alunos de pós-graduação em Fonoaudiologia. Porém, mesmo os avaliadores profissionais de T.I que não conhecem a etapa de acompanhamento auditivo na prática, mantiveram as avaliações positivas.

Dessa forma, a ausência de diferenças nas avaliações entre as categorias de juízes, indicou que a usabilidade foi bem avaliada tanto na percepção de profissionais da área de desenvolvimento de soluções tecnológicas quanto para os avaliadores que vivenciam a realidade da APS.

Por outro lado, na segunda etapa, o escore geral dos ACS foi inferior aos achados da primeira fase. Considera-se, portanto, que os profissionais da APS que participaram da primeira fase não representaram os ACS dos municípios potiguares, visto que foram observadas maiores dificuldades na usabilidade da ferramenta por parte dos participantes da segunda fase.

Embora os apps voltados para a área da saúde tenham ganhado destaque nos últimos anos, pela disponibilidade de acesso e facilidade de uso de funcionalidades que antes só eram disponibilizadas pelo computador, aspectos como disponibilidade de *internet*, acesso a dispositivos móveis voltados exclusivamente para o trabalho e treinamentos técnicos para o manuseio de ferramentas tecnológicas por parte dos profissionais de saúde, são cruciais para o sucesso da implementação da *m-health* na rotina laboral^(24)^.

Posto isso, existe uma iniciativa da saúde digital do Ministério de Saúde, o “Informatiza APS”, que tem como objetivo subsidiar a informatização das unidades de saúde e a qualificação dos dados da APS de todo o país^(7)^. Alguns apps para *smartphones* já foram desenvolvidos visando auxiliar os profissionais da APS em suas rotinas de trabalho, são eles: e-SUS AD, e-SUS território e e-SUS AB. Porém, não foi encontrada na literatura pesquisada a análise da usabilidade ou satisfação dos usuários quanto a estas soluções tecnológicas.

Em estudos brasileiros envolvendo capacitações em saúde auditiva à distância, por meio de CD-ROM, *Cybertutor* e em curso online disponibilizado na plataforma do Ministério da Saúde, verificou-se que os profissionais ACS possuíam dificuldades com informática básica. Esses dados revelaram que a adesão às estratégias de educação permanente em saúde pode ter sido influenciada pela dificuldade técnica em manusear programas de interface *web* e no *desktop* ^(6,25)^. Imaginava-se que as dificuldades dos ACS com a informática básica estivessem atreladas ao baixo acesso a computadores na APS.

Uma pesquisa realizada em 2020 apontou que o quantitativo de *smartphones* no Brasil é equivalente a mais de um aparelho por habitante, com um total de 234 milhões^(26)^. Esses dados acerca da popularização dos *smartphones* levantaram a hipótese de que as aplicações móveis voltadas para o acompanhamento auditivo poderiam possuir vantagem em relação às estratégias com implementação *web* e *desktop*. Porém, o observado neste estudo foi que mesmo com o amplo acesso aos *smartphones*, os profissionais ACS também possuem dificuldades técnicas básicas em aplicações móveis, tais como encontrar o aplicativo na tela de início do aparelho móvel, cadastrar uma senha e lembrar o e-mail.

Dessa forma, as ferramentas de aplicabilidade móvel voltadas para esse público de usuários necessitam de uma interface bem elaborada, com o objetivo de que os usuários consigam extrair o máximo possível do app, sem grandes dificuldades técnicas. A insatisfação no uso pode ocasionar uma má impressão perante o usuário e até mesmo a desistência no uso do app, o que não é desejável^(20)^.

A usabilidade está ligada à qualidade da interação de um usuário ao utilizar uma interface, seja ela de um sistema, uma ferramenta ou uma aplicação móvel. As avaliações de usabilidade buscam analisar a qualidade de interfaces de um sistema, verificando se aquele produto se mostra intuitivo o bastante, a ponto de não ter tantos ou quase nenhum tipo de falha que afete na utilização pelo usuário, com uma qualidade no mínimo aceitável. Os testes de usabilidade visam encontrar problemas de usabilidade em interfaces de acordo com a utilização que os usuários fazem delas. Neste caso, os usuários testam as funcionalidades do sistema, reportando possíveis problemas de interação em sua utilização^(20)^.

Em Bangladesh, um estudo realizou uma revisão e avaliou a usabilidade dos diversos apps de *m-Health* do país^(27)^. Os resultados apontaram sérios problemas de usabilidade identificados pelo método SUS. Os avaliadores participantes indicaram que a grande maioria das aplicações móveis voltadas para a saúde, desenvolvidas em Bangladesh, pontuaram menos para a heurística do design estético e gráfico. Os autores concluíram que o design pouco intuitivo poderia justificar a falta de adesão do uso de apps nos serviços de saúde do país.

Nos resultados do presente estudo, o design gráfico e a estética do app foram bem avaliados tanto na primeira quanto na segunda fase. Além disso, na percepção dos juízes foi obtido um maior escore para as questões relacionadas a “facilidade de aprendizagem”, com pontuação média de 97,50, o que pode estar atrelado à interface gráfica do app bem estruturada e de fácil manuseio.

Na segunda fase, os ACS participantes avaliaram a interface de forma semelhante, porém, com pontuação média de 80,71. Ao considerar o escore das questões relacionadas à interface e a baixa porcentagem de ACS que de fato utilizaram o aplicativo em suas rotinas de trabalho (8,57%), foi possível observar que embora o design gráfico do app tenha uma boa pontuação, esta variável não refletiu diretamente na adesão ao uso efetivo do app. Diante disso, a implementação do aplicativo de fato, não se mostrou atrelada à satisfação do design gráfico como visto na revisão sistemática em Bangladesh^(27)^.

A menor pontuação dentre as questões do SUS, na avaliação dos juízes da primeira etapa do estudo, foi em “satisfação do uso” com escore médio de 89,00. Embora tenha sido a pontuação mais baixa, esse escore ainda representa um valor de usabilidade excelente^(20)^. Já na segunda fase, a menor pontuação foi no que diz respeito à “facilidade de aprendizagem” com 71,00. Acredita-se que essa queda nas pontuações referentes à satisfação e facilidade aprendizagem pode ser justificada por algumas funcionalidades do app não terem alcançado as expectativas dos avaliadores, como exemplo, a ausência da opção “esqueci a senha” e a aba de “dúvidas” que não possui a possibilidade do usuário cadastrar suas dúvidas diretamente no app e as opções de respostas da TAN, durante o cadastro das crianças acompanhadas pelo app, que acabam deixando possibilidade de preenchimento errado dos dados.

Os erros de preenchimento no cadastro das crianças podem ser minimizados pela versão ADM do app, que tem acesso a todas as informações cadastradas pelos usuários. Logo, se há alguma informação incompatível dos dados em relação a TAN, o ADM tem a possibilidade de corrigi-los por meio da sua versão *web*.

Além disso, a versão deste app foi idealizada com a aba “dúvidas” em formato de fórum, como sugerido pelos avaliadores. Porém, durante o desenvolvimento da interface foi observado que a opção de inserir dúvidas diretamente no app tornaria o software mais robusto e, consequentemente, iria necessitar de mais memória livre no dispositivo para o *download.* Pensando na possibilidade dos usuários não conseguirem baixar o app por falta de memória disponível em seus *smartphones*, foi optado por deixar a aba de “dúvidas” com as perguntas fixadas e editáveis apenas pelo ADM, que esteve em contato direto com as dúvidas dos participantes.

Outros aspectos pontuados nas respostas dissertativas dos avaliadores foram os erros de digitação e sugestões da inserção de funcionalidades simples, como a opção “esqueci a senha” logo na tela de login do app. Esses tópicos apontados são de fácil correção e as melhorias poderão ser implementadas em futuras versões do app.

Em relação ao resultado da avaliação pelo NPS, em um estudo desenvolvido na África do Sul, com uma aplicação híbrida (*web* e presencial) para o acompanhamento da audição de pacientes, resultou em uma pontuação +87% no NPS dos usuários. Os autores apontaram que diante desse valor do NPS, é altamente provável que os usuários recomendem a clínica híbrida a amigos e familiares^(28)^.

Levando em consideração os resultados da avaliação no NPS neste estudo, +100% dos avaliadores da primeira etapa provavelmente recomendariam o uso do app para outros usuários, mantendo-se na categoria de ‘promotores’ em relação à avaliação do NPS. Já em relação aos participantes da segunda fase que apontaram +48,58% no NPS, demonstram que a ferramenta embora não apresente sérios problemas de usabilidade, ainda não seria tão recomendada para outros usuários quando comparado aos participantes da primeira etapa.

Em um estudo brasileiro com foco na capacitação em saúde auditiva dessa categoria, os profissionais que desistiram do estudo apontaram como principais motivos, a rotatividade de profissionais e cargos, falta de participação dos gestores e a elevada demanda de atividades voltadas aos ACS^(4)^. A falta de clareza sobre as atribuições do ACS pode provocar sobrecarga na jornada de trabalho e, por não possuírem um plano de carreira definido, estes profissionais acabam realizando tarefas diversificadas e sem padronização. Este é um ponto importante para a implementação de novas ferramentas, pois além de adequada usabilidade, a motivação dos profissionais para o uso efetivo é essencial.

A satisfação do uso do app na rotina de trabalho foi prejudicada, uma vez que no período deste estudo, as funções dos ACS foram redirecionadas para apoiar ações dos profissionais de saúde voltadas à pandemia da COVID-19, como a testagem intensa e a campanha nacional de vacinação. Assim, apesar dos resultados promissores, o número reduzido de ACS que utilizou o app na rotina de trabalho de forma efetiva na terceira etapa não permite generalizar os dados obtidos, sendo esta uma limitação do estudo.

## CONCLUSÃO

O protótipo do app para *smartphones* “Agente Escuta”, desenvolvido para auxiliar a etapa de acompanhamento auditivo e de linguagem na APS, apresentou boa usabilidade segundo os participantes potiguares, sendo que 90% dos juízes e 60% dos ACS dariam publicidade positiva ao produto. Os ACS que utilizaram o app na rotina, concordaram com a importância e relevância de se realizar o acompanhamento auditivo, indicaram influência positiva do Escuta Game na revisão dos conteúdos sobre saúde auditiva e demonstraram interesse em continuar utilizando o app na rotina de trabalho.

Melhorias deverão ser implementadas para as próximas versões do aplicativo ou na proposição de outras soluções tecnológicas para o público-alvo, de acordo com as sugestões dos avaliadores deste estudo.
